# Valuing child and adolescent health: a qualitative study on different perspectives and priorities taken by the adult general public

**DOI:** 10.1186/s12955-021-01858-x

**Published:** 2021-09-23

**Authors:** Philip A. Powell, Donna Rowen, Oliver Rivero-Arias, Aki Tsuchiya, John E. Brazier

**Affiliations:** 1 School of Health and Related Research, University of Sheffield, Regent Court, 30 Regent Street, Sheffield, S1 4DA UK; 2 National Perinatal Epidemiology Unit (NPEU), Nuffield Department of Population Health, University of Oxford, Oxford, UK; 3 Department of Economics, University of Sheffield, Sheffield, UK

**Keywords:** EQ-5D-Y, Health-related quality of life, Perspective, UK, Qualitative, Valuation exercise, Youth health state valuation

## Abstract

**Background:**

Quantitative health preference research has shown that different “perspectives”, defined here as who is imagined to be experiencing particular health states, impact stated preferences. This qualitative project aimed to elucidate this phenomenon, within the context of adults’ valuation of child and adolescent health states.

**Methods:**

Six focus groups with 30 members of the UK adult public were conducted between December 2019 and February 2020 and analysed using framework analysis. Each focus group had two stages. First, participants individually completed time trade-off tasks and a pairwise task (mirroring a discrete choice experiment without duration) for two EQ-5D-Y health states, assuming a series of perspectives: (a) themselves at current age; (b) another adult; (c) 10-year old child; (d) themselves as a 10-year old child. Second, a semi-structured discussion explored their responses.

**Results:**

Participants’ views were often heterogeneous, with some common themes. Qualitatively, participants expressed a different willingness to trade-off life years for a 10-year old child versus themselves or another adult, and this differed by the health profile and child imagined. The same health states were often viewed as having a different impact on utility for a 10-year old child than adults. Imagining a 10-year old child is difficult and there is variation in who is imagined. Participants found answering based on their own—adult perspective most acceptable. There were no strong preferences for prioritising child health over working-age adults’ health.

**Conclusions:**

If an adult sample is used to value child- and adolescent-specific health states it is important to consider the perspective employed. Members of the adult public provide different responses when different perspectives are used due to differences in the perceived impact of the same health states. If adults are asked to imagine a child, we recommend that sampling is representative for parental status, since this can affect preferences.

**Supplementary Information:**

The online version contains supplementary material available at 10.1186/s12955-021-01858-x.

## Background

Methodology for measuring and valuing health benefits in adult populations is well established [1], including detailed guidance from international health regulatory agencies, such as the National Institute for Health and Care Excellence (NICE) [2] and the Pharmaceutical Benefits Advisory Committee (PBAC) [3]. However, this is not the case for measuring and valuing health benefits in children and adolescents, where there is a lack of detailed guidance [4].

Child and adolescent preference-based measures are designed to measure and value the health of young people. For example, self-reported EQ-5D-Y-3L is for use in children and adolescents aged from 8 to 15 years [5–7]. Child- and adolescent-specific preference-based measures differ to adult measures in important ways in how they measure health [8]. One crucial difference is their value sets for economic evaluation. Methodological decisions for the valuation of preference-based measures may differ for child and adolescent versus adult measures [9]. Important methodological considerations are: whose preferences (e.g. adults’ or children’s); what perspective (e.g. for yourself or another, such as a child); and which elicitation technique (e.g. time trade off [TTO] or discrete choice experiment [DCE]), including (where relevant) methods to anchor onto the 1–0 full health-dead scale [4]. Whilst some of these methodological decisions are normative, empirical research can help inform the selection of appropriate methods and enable better understanding of the impact on the value sets arising from different methodological choices.

Preferences for child and adolescent health states can be elicited from adolescents or adults (since younger children cannot meaningfully complete elicitation tasks [10]), and evidence shows that adult and adolescent preferences differ when valuing health states for themselves [11–14]. Adult preferences can be argued for based on considerations such as: adults (i.e. ≥ 18 years old) represent the voting and tax-paying public and their views should determine allocation of publicly funded healthcare resources; adults are better suited to participate in preference elicitation tasks that can be cognitively demanding; and it is more ethically acceptable to ask adults to choose between hypothetical scenarios involving death. However, whilst adults may have a greater understanding of the tasks, crucially they may not understand or be able to imagine what it is like to experience child and adolescent health states and how they impact younger individuals (see [4, 15] for a more detailed overview of the arguments for and against adult and adolescent preferences). Knowledge of the general public’s preferences (who are often the target sample in health state valuation studies) on who they think should value child and adolescent health states is scarce.

Adult values for child measures can be elicited by different approaches on whose health is valued, namely different “perspectives”. These include, but are not limited to: (a) health state for themselves at their current age (i.e. “own—adult”); (b) health state for another adult (i.e. “other—adult”); (c) health state for another child at a specified age (i.e. “other—child”); and (d) health state for themselves as a child at a specified age (i.e. “own—child”). The choice of perspective can impact on elicited preferences [16, 17]. The own—adult perspective can be advocated for on the basis that it is comparable with the methods used to generate value sets for adult measures. However, the health state they are imagining may differ to the health state that is being measured in an instrument used with children and adolescents. For example, usual activities would typically differ by age, and may differ in relative importance to the overall utility of the health state. Alternatively, adults could be asked to imagine the health state in the context of a 10-year old child (other—child), for example, but potentially it may matter which child they imagine (e.g. their own child vs. another child) [18]. Adults can also be asked to imagine the health states for themselves as a child (own—child), which could be prone to recall bias (as they may not accurately recall themselves as a child) and could be influenced by their views, for example around child health and childhood [4]. A deeper understanding of how members of the public respond to different perspectives when valuing child and adolescent health is of use in contributing to this debate.

Different techniques can be used to elicit preferences, but the combination of elicitation technique and perspective can affect values. Research using visual analogue scales (VAS) found that values elicited using an own—adult health perspective were higher than values elicited using an other—child perspective [16], whereas research using TTO found the opposite [17]. TTO asks respondents to trade life years for improved health, whereas VAS involves no such trade-off (the same is true of DCE tasks where duration is not an attribute), and hence the different results may occur if participants are less willing to trade off a child’s life than to trade off their own life. However, the reasons for this pattern of responses, and how it relates to preferences for prioritising child versus adult health, are currently unknown. In particular, often studies compare responses between own—adult and other—child conditions [16, 17], and so are not able to ascertain the degree to which differences are due to the subject (i.e. adult vs. child) or perspective (i.e. own vs. other) (for an exception see [19]).

Past research has focussed upon quantitative surveys examining whether elicited values vary depending on methodological decisions, for example by whose preferences and the perspective used, elicitation technique, and the health state classification system [12, 14, 16, 17, 20]. Whilst it has been established that these methodological choices impact on values, what has not been more widely explored are the reasons why values differ and the preferences lay people have for how child and adolescent health states should be valued. Given that they collectively bear the costs of healthcare and may benefit (either indirectly or directly) from health technologies, understanding what the general public think about the different approaches used to value health for children and adolescents is of potential value to researchers and decision-makers. This motivation for public consultation can be considered analogous to the “payer perspective” argument to justify the use of adult general public samples in health state valuation exercises (e.g. [18]) and is consistent with public and patient involvement and engagement initiatives widespread across health research [21]. Understanding what the public think about these issues can impact on the appropriateness of the methodological choices that are made and their acceptability to policy makers, and thus can enable researchers to account for these factors in valuation study design.

In understanding the public’s views on health state valuation for children and adolescents, it may also be of value to explore what individual difference factors relate to, or underlie, these views. For example, whether parental status plays a role, and whether wider views around child and adult health, including relative prioritisation in health resource allocation, relate to variation in the public’s opinions on child health valuation.

The aim of this qualitative research is to examine and better understand the way that a system of perspectives; elicitation technique (TTO and DCE); and wider views around child and adult health, including whether either should be prioritised in resource allocation, impact on the way members of the adult general population value child and adolescent health states. The work sought to: (1) better understand how perspectives impact on values for child and adolescent health states elicited from members of the UK adult general population, and (2) understand how values are impacted by general attitudes to child and adult health and people’s prioritisation of child versus adult health. This is informative for the design of future studies valuing child and adolescent health.

## Methods

### Recruitment and participants

Six focus groups with five members of the general public in each (*N* = 30) were recruited via Accent Market Research, Sheffield, UK. Six focus groups are typically sufficient to produce reasonable data saturation (with up to 90% of themes uncovered, [22]). Recruitment was specified to achieve a mixture of gender, age, and whether the participants had children aged under 18 years. Information sheets and consent forms describing the study were given to participants in advance. Participants were paid £40, in accordance with standard market research rates.

### Focus group procedure

The focus groups were held at the University of Sheffield, in the evening between December 2019 and February 2020. Upon arrival, participants were greeted by a researcher who carried out informed consent procedures. Two experienced researchers (PP and DR), with expertise in qualitative research and health state valuation, facilitated the focus groups.

**Fig. 1:**
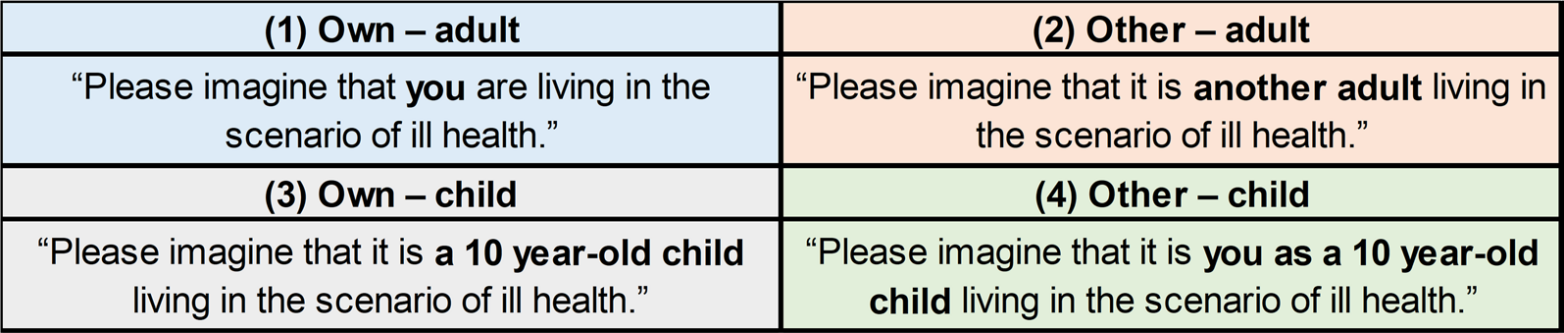
System of “perspectives” and accompanying text used in the study

Following an introduction to the focus group, participants completed a background questionnaire on their gender, age, and whether they had children under 18 years old, and completed the EQ-5D-Y-3L [5–7] for their own health to establish familiarity. One researcher (PP) introduced and explained the TTO exercise [23], and the other researcher (DR) demonstrated the TTO exercise using a visual prop (from the MVH protocol [24]). Participants then completed TTO exercises for two EQ-5D-Y-3L health states individually, where participants chose between living in the health state for 10 years before dying or living in full health for a shorter amount of time, with the latter decreasing in 6 month decrements (from 10 to 0 years). Participants then completed a choice task formatted to mirror a pairwise DCE without duration, where participants were asked to choose between living in two EQ-5D-Y-3L health states for 10 years before they died. Participants completed the TTO and DCE tasks four times using a system of “perspectives” (Fig. 1), in this order: (a) own—adult; (b) other—adult; (c) other—child (at 10 years old); (d) own—child (at 10 years old). Participants independently completed the valuation exercises, with understanding checked by the researchers. Participants were able to ask questions of the researchers throughout. All tasks were completed on paper in hard copy. An example questionnaire for the own—adult perspective is provided in Additional file 1, Appendix A.

When all of the exercises were complete, a semi-structured discussion on understanding and valuing the health of others (of different ages) was facilitated. The discussion concentrated on: (1) exploring participants’ understanding and interpretation of the health states; (2) exploring differences across the system of perspectives and task; and (3) exploring “solutions” (or participants views about resource allocation and being informed). A semi-structured topic guide was produced to guide the discussion (Additional file 1, Appendix B). The focus group was audio-recorded and lasted for an average of 84.8 (*SD* = 1.5) minutes, with approximately 50.7 (*SD* = 3.5) minutes on the discussion.

### Health states

**Fig. 2:**
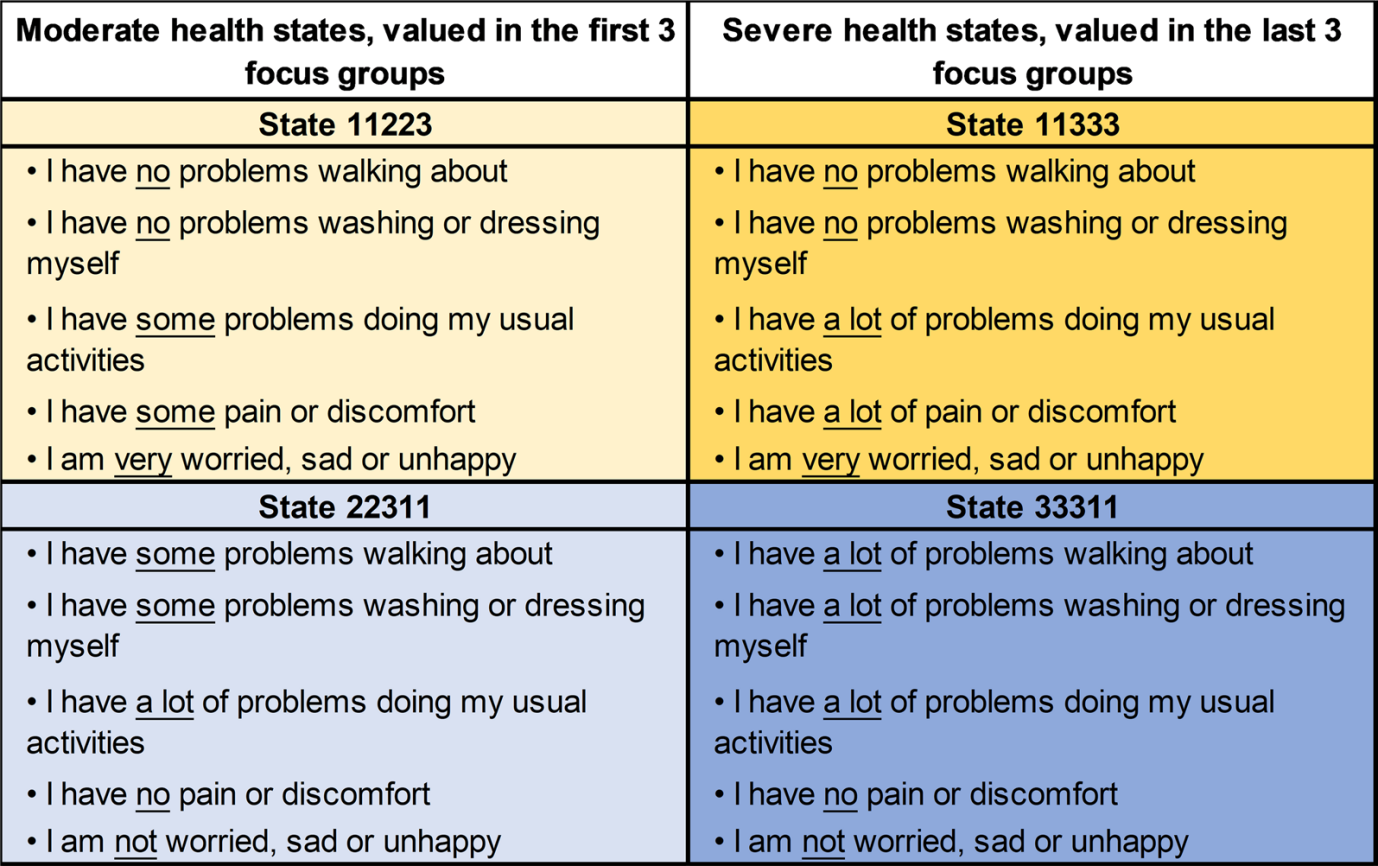
EQ-5D-Y-3L health states included in the focus groups, worded for own—adult perspective

In order to facilitate the health state valuation exercises and explore the influence of perspective, two different EQ-5D-Y-3L [5–7] health state profiles were selected (labelled “health state A” and “health state B”). These profiles consisted of five dimensions of health defined by the EQ-5D-Y-3L classification system (mobility; looking after myself; doing usual activities; having pain or discomfort; and feeling worried, sad or unhappy), with three levels of severity (no problems, some problems, a lot of problems), represented by digits 1–3. One health state was more aligned to mental health and the other to physical health to enable discussion on the impact of mental versus physical health on valuation using lay terms, though it is acknowledged these are simplifications. To explore the effect of severity, the first three focus groups received moderate health states (EQ-5D-Y profiles 11223 and 22311) and the last three focus groups received more severe health states (EQ-5D-Y profiles 11333 and 33311). The health states are presented in Fig. 2.

### Analysis

The sociodemographic and health characteristics of participants were summarised. Responses to the TTO and DCE tasks were summarised, but not assessed statistically due to the sample size.

The discussion element of the focus groups was transcribed verbatim for analysis and checked for errors. The transcripts were analysed using framework analysis [25], in six stages [26]:

1. *Familiarisation* Two researchers (PP and DR) independently read and re-read the transcripts, while listening to the audio recordings, to increase familiarity with the data.
2. *Coding* Two researchers (PP and DR) independently assigned codes (i.e. summary labels) to 50% of the transcripts in hard copy using the margins. Coding was informed by a priori themes included in the topic guide (Additional file 1, Appendix B) and emerging themes from the data.
3. *Developing the framework* The researchers (PP and DR) met to discuss their coding and consensus was reached on a provisional analytic framework featuring a set of codes, grouped within draft categories. The primary researcher (PP) then applied the draft framework to the remaining transcripts, while noting any new codes or ideas that emerged. The second researcher (DR) read the remaining transcripts and coded any new themes. The two researchers (PP and DR) met to discuss and refine the draft framework and its fit to the data, before agreeing upon a final framework.
4. *Indexing* The final framework was applied (indexed) on fresh versions of the transcripts by the primary researcher (PP), using Nvivo v12 (QSR International Pty Ltd., 2018). The second researcher (DR) checked two randomly selected manuscripts for agreement with indexing. Three themes that had a low level of coverage in the data (less than five separate instances) were merged with other related themes.
5. *Charting* Microsoft Excel was used to summarise the indexed data in a matrix, with one row per code, and one column per participant, with a separate sheet for each category. Each cell in the matrix was then populated using verbatim data from the transcripts.
6. *Interpretation* All researchers met to discuss and agree on the final interpretation of the data, including descriptive memos for each of the themes, codes contained within each category, and supporting data (including disconfirming cases).

This research received ethical approval from the corresponding author’s host research institution.

## Results

### Sample characteristics

Each of the six focus groups consisted of five participants, with no no-shows. Of the 30 participants, 17 were male and 16 had children under 18 years old. Participants’ ages ranged from 18 to 69 years (*M* = 44.4, *SD* = 14.4). Each focus group involved participants with a range of age, gender, and current parental status. EQ-5D-Y responses to the dimensions varied and no participants reported having a lot of problems in any dimension. The percentage of participants reporting some problems was 16.7% for mobility; 0% for looking after myself; 23.3% for doing usual activities; 60% for pain or discomfort; and 36.7% for feeling worried, sad or unhappy.

The TTO and DCE results reveal some patterns that are useful when interpreted alongside the qualitative findings. First, in both tasks, participants typically preferred the “physical health” state (states 22311 and 33311) over the “mental health” state (states 11223 and 11333), with one exception (own—adult perspective, DCE, severe health states). Second, in the TTO tasks, participants were generally willing to trade more life years to avoid more severe health states, with two exceptions (own—child perspective, mental health state; other—child perspective, physical health state). Third, within-group mean TTO values fluctuated by perspective, with participants least willing to trade off life years within each health state for the other—child perspective, and particularly so in a physical than mental health state.

### Qualitative results

**Fig. 3:**
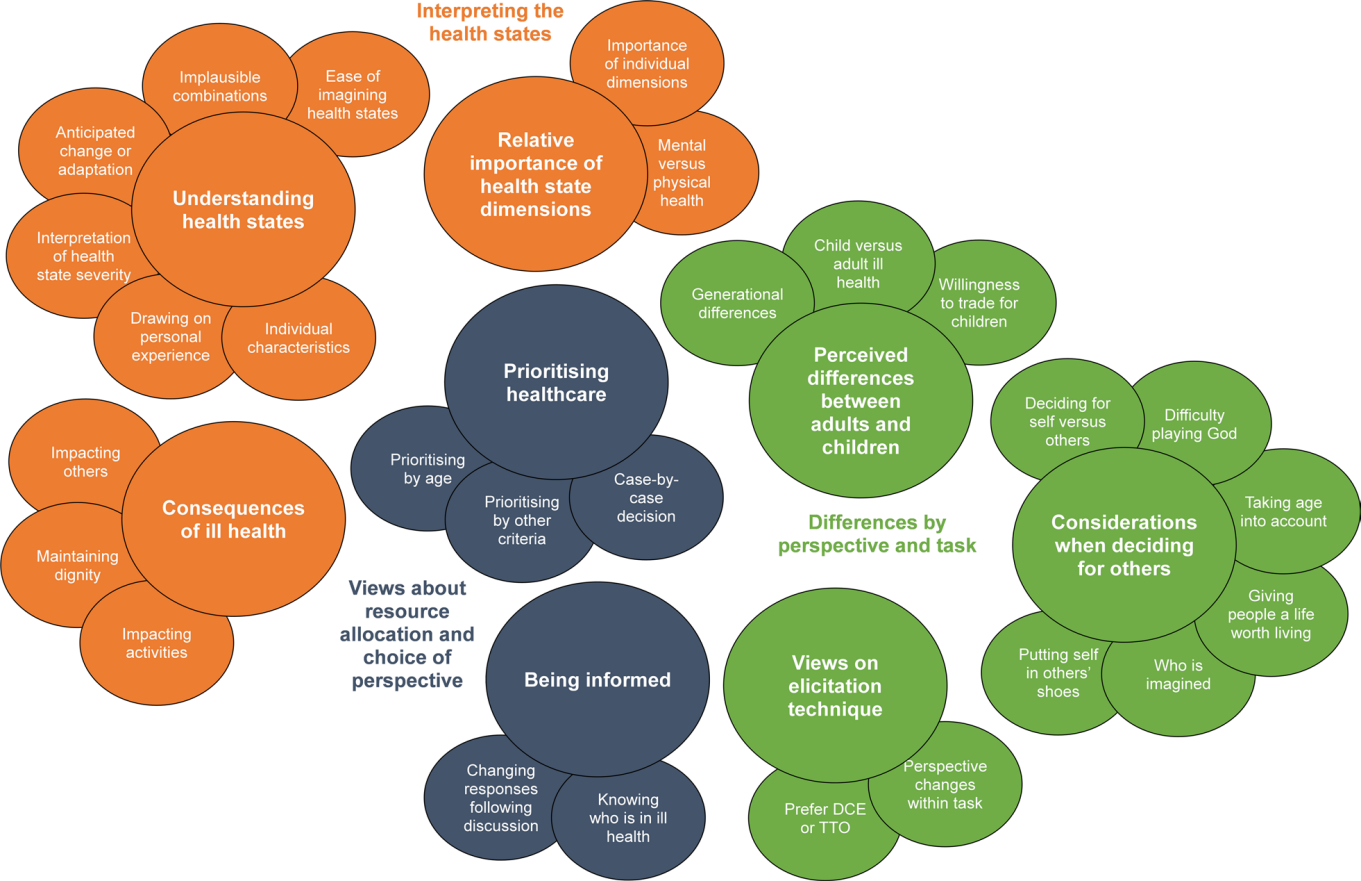
Thematic framework. *Notes*. Themes are likely to have bi-directional relationships (not visualised on the diagram)

Twenty-seven themes emerged from the framework analysis, categorised within eight categories that mapped onto the three superordinate topics of interest outlined in the topic guide: (1) interpreting the health states; (2) differences by perspective and task; and (3) exploring participants’ views about resource allocation and being informed. The themes are graphically outlined in Fig. 3 and their data coverage is summarised in Additional file 1, Appendix C. The analysis showed good saturation, with no new themes first indexed in the final two focus groups (and only one in the latter three focus groups; see data saturation table in Additional file 1, Appendix D). While discussed separately, many of the themes are likely to be interrelated, with bidirectional relationships with one another. Unless otherwise indicated, results are synthesised and the discussion is applicable for all perspectives.

#### Topic 1: interpreting the health states

##### Category 1: understanding health states

*Ease of imagining health states* Participants differed in the extent they found the health states easy to imagine. Most found it difficult, stating that they did not actually say what was the matter (e.g. backache) and would have benefited from more concrete information: “*…with them health states you gave. It’s. It’s kind of quantifying or putting a bit more substance to those and understanding what the problem is a bit more.*” (Participant 2 [P2], Focus Group 3 [FG3]). Those who found the states easier to imagine typically had some prior experience of them, either personally or via people they knew.

*Implausible combinations* Some participants struggled with a realistic imagining of the combinations of proposed levels of the health state dimensions, noting that if they were not mobile or able to wash and dress themselves then they would also be unhappy: “*I kind of find B more not believable, because of the ‘I’m not worried, sad, or unhappy’. If you kinda can’t do much of that above, you’re gonna be a bit sad and unhappy somewhere surely*.” (P1, FG1). This implausibility also had an impact on the perceived severity of health states, with participants stating, for example, that severe pain can’t be that bad because they could still walk around. Further ambiguity arose in the interpretation of “usual activities”, which could incorporate walking.

*Interpretation of health state severity* There were differences of opinion in the interpretation of the health state dimension levels and their severity. Participants did not like the use of vague quantifiers and wanted more examples indicating how severe the problems were: “*Erm, like you’ve got ‘some’ so what, what does ‘some’ mean? What’s that equate to?*” (P3, FG3). Participants differed in the extent they perceived the health state as severe and this impacted their decisions. As expected, the first three focus groups typically interpreted the health states as being less severe than the latter three focus groups.

*Anticipated change or adaptation* Despite the task instructions, a common idea was the health states could, or would, change over the 10-year period. This could be through directly treating the problem or adapting to it: “*You know, like you don’t ever see people moaning and things like that, so you can adapt your life like that.*” (P4, FG1). Further, there were differences in anticipated change as a function of health state dimension, with pain for example seen as controllable with medication, and mental health seen as harder to change.

*Drawing on personal experience* Participants frequently drew on personal experience, both to help them understand the health states and inform their decisions. Experience ranged from that of themselves, close others, and people they knew, to professional roles (e.g. working in special needs education): “*I’ve got experience with special needs children in some special schools (…) even though they’ve got a certain disability, they still live virtually a full life and enjoying it like any other.*” The experience mentioned was frequently of older family members in ill health. In drawing on personal experience, the participants often related the health state descriptions to known medical conditions, such as dementia or anxiety disorders.

*Individual characteristics* It was acknowledged that individual differences may play a systematic role in how people responded to the health states and the decision tasks. This included age of the respondent, whether they had children, and their personality or coping styles: “*I think if you’re in that age bracket then you’d see it differently.*” (P3, FG5).

##### Category 2: Relative importance of health state dimensions

*Mental versus physical health* Much of the discussion around the health states involved a comparison between problems related to mental versus physical health. Many participants viewed dimensions that they related to mental health as more important in considering the severity or impact of the health state than the physical health dimensions. This related to the theme of giving people a life worth living and minimising suffering. This was true of themselves and imagined others, including children: “*And the way I was looking at this was not the physical, it’s the mental (P3: torture) which then. Yeah.*” (P1, FG6). However, there was a non-trivial minority of people who saw physical health as more important, who also tended to lead active lives.

*Importance of individual dimensions* In addition to the debate over mental and physical health, frequently participants focused on a particular dimension within a health state, such as one aspect of being worried, sad or unhappy: “*It was the very worried bit (…) Everybody’s a bit sad and down for various reasons throughout life but the very worried bit sort of if you’re worrying all the time.*” (P2, FG1).

##### Category 3: Consequences of ill health

*Impacting activities* A consequence of ill health that was mentioned by a subset of participants was the impact of ill health on being “active”, and doing activities that they currently derived fulfilment from: “*…myself and the family as a whole, we’re all really active and that I think, that would be something that I would struggle to come to terms with not being able to do the things that I’m used to doing.*” (P2, FG4).

*Impacting others* When considering the health states (from multiple perspectives), participants frequently thought about the impact of ill health beyond the person experiencing it. In particular, this included the effect on family members and close others. A strong trend was the concept of retaining independence and not burdening others, particularly loved ones: *“…I also don’t want to burden her with, you know, looking after me because I’ve lived that myself, do you know what I mean?*” (P3, FG6). When thinking about the effects of ill health on children, some participants emphasised that children and adults are linked via family units and so they thought about the impact on both. Some participants indicated that they would be prepared to live in ill health to be around for others (e.g. to look after their own children): “*I would be pretty much willing to put up with any kind of ill health to be around for my kids.*” (P5, FG4).

*Maintaining dignity* An important consequence of ill health for some participants was maintaining dignity. This included, for example, feeling embarrassed in having others, including loved ones, looking after oneself physically: *“…it is down to dignity, I wouldn’t have wanted my, me partner, I thought about her and me kids, I wouldn’t want them to see me like that (…) you don’t want your kids washing or bathing you, do you?*” (P2, FG5).

#### Topic 2: Differences by perspective and task

##### Category 4: Perceived differences between adults and children

*Generational differences* One theme raised by participants was generational differences in relation to ill health. This included a perceived worse ability to cope with mental health issues and stress in the younger generation: “*They’re a bit fluffier now aren’t they (P1: snowflakes), do you know what I mean? That’s how it’s changed!*” (P4, FG1). Furthermore, a few participants referred to the greater availability of support and assistive technology for people experiencing ill health now, compared to in the past. This discussion was relevant to the perspective of participants thinking about themselves as a 10 year old and whether this was framed as now or in the past.

*Child versus adult ill health* A significant proportion of the discussion centred on the perceived difference between child and adult experiences of ill health. Many participants assumed that the 10-year old child had been born with the health condition, rather than acquired it, though the reverse was seen as more likely for the other adult: “*Because I thought, you know, at 10 year old you wouldn’t know any different, if you couldn’t walk you, you know, that has always been something that’s kind of been there (yeah) ant it?*” (P3, FG6). Participants disagreed on whether the health states were worse, just as bad, or better for children to live through than adults. Some participants thought that the states of ill health might be worse for children, and reasons provided included that they thought an adult had greater capacity to deal with ill health, that they did not like to see a child unhappy, that the physical implications would be greater given that children are typically more active, and that children were more vulnerable: “*State B is probably worse for children just because of that timeframe (…) Adults have already had the time to experience things like. Well, a, a healthy childhood, whereas the children haven’t.*” (P4, FG2). In contrast, other participants stated that ill health was worse for adults and described children as being more resilient and just getting on with it despite ill health: “*I worked with children with special needs and the ability is far better than an adult to overcome these things. They stay a lot more cheerful.*” (P2, FG1). Other arguments for this position included that children have existing support unlike adults, particularly within their family, that there is more stigma around adult mental ill health, and that adults have more responsibilities including caring for children.

*Willingness to trade for children* On the whole, participants were generally less willing to trade life years for children than adults: “*When it came to the child I found that quite hard. Erm, and I, I put that they should live for the 10 years regardless for everything.”* (P2, FG1). This was particularly the case for physical states of ill health and less so for problems in the dimensions of worried, sad, or unhappy and pain or discomfort (the mental health state) were perceived as severe enough. The latter was motivated by a desire to minimise suffering for the child: “*I think I did shorten their life (laughs). Because you think (laughs), and I’ve got two kids and that’s awful (laughs). But I think the thought of a chi, you know the thought of that of being, saying you were constantly in hospital and they were miserable and they were really struggling and they were in a huge amount of pain.*” (P6, FG4).

##### Category 5: Considerations when deciding for others

*Putting self in others’ shoes* When deciding for others, participants described an empathic process of attempting to put themselves in others’ shoes, and using that to decide what the other adult or child might want. Participants also discussed the difficulties of putting themselves in others’ shoes in some cases: “*I think for me, I put myself in the, in the child shoes and I think if you ask any child would they take any health state over living for a shorter period.*” (P2, FG4).

*Deciding for self versus others* Some participants treated others, and particularly other adults, like themselves, giving the same answers. Other participants stated that they knew what they could put up with, and thus they were more willing to either trade life years or “put up with” health states, depending on their preferences: “*I was thinking there is only so long I’d be able to put up with it before that’d be it, I’d have had enough personally. Whereas another human being it would just be difficult to see their life end.*” (P5, FG6). Participants noted a conflict between what they wanted and what they thought other people would want for themselves, including a desire to keep close others alive: “*…it’s selfish because you want them to still stay. So you’ll be like you know in any state I want to keep them. But then, them probably thinking (…) same as us, you know, I’d rather have like 6 months of full health than 10 years in a state like that.*” (P1, FG5). Ultimately, participants felt most happy in researchers using answers from the own—adult perspective.

*Who is imagined* There were marked differences in who participants imagined when doing the valuation tasks in different perspectives, and this was raised as a key factor in motivating decision-making. Often people thought about another family member, including children. For the other adult, it was often an older family member: “*I think on that one as well it depends who you’d got kind of in mind. I were kind, for myself, I were picturing like me nannan or someone like that.*” (P5, FG5). Participants who were parents noted that they may respond differently due to thinking about their own children (vs. others): “*But then again, well, it depends, is it your child? Cause I think that would be a different response to somebody else's child.*” (P1, FG4). One person took a wider perspective (for another adult) of the whole of society. Participants were open that their answers would be different if they thought about someone else, and a few participants were explicit about wanting to prioritise their own family above others.

*Difficulty playing God* Participants often expressed that they found the topic of deciding for others difficult, with a particular emotional impact of thinking about children (suffering) in ill health. Frequently, participants would prefer to make no decision than to make a call about shortening the lives of others, or 'playing God': “*It’s like playing God in’t it? I mean how these people do that I don’t…*” (P2, FG1).

*Taking age into account* When thinking about other perspectives, some of the participants thought very carefully about the age of the person imagined in that scenario, thinking about their life stage, how much to trade, at what age they would die, and what they would be doing during that period of life: “*I started doing it well if, if the child dies when they are 17, let’s say. Is that better than them dying when they are 10?*” (P4, FG2). It was noted that age was specified for the child, but not the other—adult perspective. Participants had some debate whether their view may be different for a child of a different age (e.g. 15).

*Giving people a life worth living* A common theme was wanting to minimise the suffering of others, especially children, and give people a life worth living: “*I don’t want to see anyone suffer*” (P5, FG6). This was a key factor in decision-making. Participants generally felt that being worried, sad, or unhappy, and/or in pain the mental health state represented a greater state of suffering than the other health dimensions. This desire to minimise suffering counteracted participants’ unwillingness to trade life years for others, especially for children: “*I wouldn’t want a child to be in pain, and worried or sad or unhappy, I wouldn’t want that*” (P1, FG4).

##### Category 6: Views on elicitation technique

*Prefer DCE or TTO* Participants were split on whether they preferred doing the TTO or DCE tasks. Arguments for the DCE was that it was easier and less complicated and did not involve “gambling with someone's life”: “*The last question is the easiest because it is either one or the other there are no grey areas.*” (P1, FG6). Arguments for the TTO was the increased granularity that enabled them to provide more detailed information (i.e. cardinal rather than ordinal preferences): “*I think the first one as well, I think there’s just more context to it (…) it’s not 10 years with that or nothing.*” (P5, FG5).

*Perspective changes within task* The perspective taken had a potential differential effect on the TTO and DCE tasks, with participants viewing their responses as being more stable in the DCE tasks than the TTO tasks across different perspectives: “*So I think that for me, A and B [DCE], I’ve been resolute throughout. I have always known the answer. Whereas with these ones [TTO] it’s, er, yeah.*” (P4, FG2). Some people were non-traders on the TTO and thus their TTO utilities did not differ by perspective.

#### Topic 3: Exploring participants’ views about resource allocation and being informed

##### Category 7: Prioritising healthcare

*Prioritising by age* The idea of prioritising healthcare or healthcare funding by age was mixed. While many would prioritise children over adults to receive the same gain in length and quality of life from healthcare, particularly if it was a forced choice, other participants pushed back against this, and some thought that ill health, or an earlier death, is worse for adults and so would prioritise them over children: “*You’d help a child more than you would help an adult, wouldn’t yer? Especially a 10 year old child.*” (P3, FG4); “*I think that would be my reasoning behind the adult they have got probably (…) more of a family that would potentially be impacted.*” (P1, FG4). An alternative, common preference was that neither children nor adults should be prioritised, rather that there should be ‘equal’ treatment regardless of age, need, or any other factors: “*And that’s not what NHS is about is it? It is about non-discriminatory treatment for anybody, and that has, that has to be start don’t it, for me.*” (P2, FG5). When considering prioritising by age, perhaps the strongest trend, advocated by many participants, was a “fair innings” argument [27]. Participants were much more willing to prioritise younger people against the elderly (e.g., aged 70 or 80 years and above): “*I would pick a child over an older person cause I think they’ve had 70 years. And that’s quite a long, you know, if you get to 70 I think, you’re doing quite well (P3: A ‘good innings’ is what we say).*” (P4, FG3).

*Prioritising by other criteria* Participants often brought up other criteria that may be used to prioritise treatment over and above, or instead, of age. This included prioritising those who would most benefit, resultant quality of life, or other criteria, such as responsibility for the illness, the patients’ contribution to society, or how long the patient has been waiting for treatment: “*The only thing that would change my, like make me decide either way is quality of life (…) if they had a rubbish quality of life, that would be my deciding factor.*” (P3, FG1).

*Case-by-case decision* A few participants did not want to decide on prioritising healthcare without more information. They said that they could not apply a blanket rule and would rather make case-by-case decisions: “*I think each decision would be on, on a case by case. It’d have to be because you couldn’t, you couldn’t generalise something like that.*” (P4, FG1).

##### Category 8: Being informed

*Knowing who is in ill health* Participants struggled with the idea of wanting to know whether or not the health state was describing that of a child and that the results of the tasks were being used to generate values for child and adolescent health states. Arguments for knowing were that it would make responses more accurate and people have a right to know: “*…obviously my answers were different, so. Just for the hypothetical, you know, put yourself in that situation I think you do need to know.*” (P4, FG4). Arguments against knowing included that whom participants would be thinking about would vary, and that knowing it referred to a child could unduly influence responses due to emotion and bias.

*Changing responses following discussion* Some participants stated that they would want to change their responses following the group discussion: “*…now that we’ve had a discussion, I, I might! I might change it. Cause I, I probably hadn’t looked at the depression part. Er, and give it as much importance.*” (P5, FG2). This raises the issue of the power of deliberation prior to responding and casts doubt over the stability of participants “stated preferences”, raising methodological questions.

## Discussion

This paper has presented findings from six focus groups conducted with a sample of the UK adult general population examining the impact of a system of “perspectives” and elicitation technique on their preferences for child and adolescent health states. We find that members of the adult general population provide different responses when different perspectives are used due to differences in the perceived impact on utility. Our findings further demonstrate that imagining and responding for others, particularly a 10-year old child, is difficult and there is variation in who is imagined. Overall, we did not find support for prioritising child health over working-age adult health, though there was some support for a “fair innings” argument.

In this sample and elicitation exercises used, problems in dimensions associated with mental health (worried, sad, or unhappy; pain or discomfort) were often, but not always, viewed as worse by participants than problems in dimensions associated with physical health (mobility, self-care), especially for children. This is consistent with emerging findings from EQ-5D-Y valuation studies, whereby the former two dimensions received higher weights than the latter [28]. These preferences interacted with participants’ willingness to trade life years for children. That is, while many participants were unwilling to shorten the lives of children (than their own life or that of another adult), they indicated that they were more prepared to do so if the health state was perceived as severe enough that the child was “suffering”. Greater suffering in this instance was viewed as having higher levels of worry, sadness, or unhappiness and/or being in pain. The implication of this is that an unwillingness to trade-off survival for children is not absolute, but may depend on the relative dimension levels and perceived severity of the health profile by participants.

The study provides evidence around how participants approach the elicitation tasks, and reinforces concern communicated elsewhere around interpretation, where participants expressed misunderstanding that the health states could change due to medication or health technologies, or, for example, replacing one’s usual activities with different activities [29]. Of concern is that respondents effectively changed dimensions that they viewed as implausible in combination with other dimensions. This raises concern over the extent to which participants in elicitation tasks are actually valuing the same health state as described.

A key factor that affected interpretation of the health states and decision-making was participants’ personal experience. Those participants who reported finding the health states easier to imagine were those with some experience of it, either directly or vicariously. When answering for other people, participants found it easier, and were more comfortable, responding to the valuation tasks if they had been exposed to other people with similar health problems (or related experiences).

Participants viewed ill health as different for adults and children, even when described with the same health profiles. The study raised a novel finding that some participants regarded the health state from the other—child perspective as ill health that had been apparent from birth, whereas for the adult perspectives they viewed this as a change from their normal health, and that this had different implications for perceived adaptation and ability to cope. Another key finding is that participants commonly raised the view that children were more resilient and had an existing support network unlike that of adults, and hence ill health may not have as large an impact on the child as for an adult, though the opposing view was also raised. Similar heterogeneous findings have been reported elsewhere [30]. There was disagreement around whether ill health had more or less impact on utility for children than adults, and this may contribute to at least some of the differences in utilities elicited using different perspectives in large scale surveys, depending upon the sample characteristics.

Our results indicate that there was minimal consensus around whether participants thought that adult participants valuing child and adolescent health states should be told whether the health state is experienced by, or will impact resource allocation decisions for, children. However, it was raised that the use of the child perspective and hence knowing that the state was for a child meant that the preferences that were elicited could potentially include emotion and bias. Methodologically, it is a point of normative debate over whether health state values should only reflect the severity of the health state (as perceived by participants) for the person experiencing that state (see for example [31]), or whether they should also reflect participants’ perceptions, emotional reactions, or wider societal views that may go beyond the perceived severity of that state. Accordingly, the degree to which switching from an adult to child perspective alters the emotion and breadth of information involved in the valuation process, and thus alters participant decision-making, is an interesting avenue for future enquiry.

It was also raised throughout the focus groups that people did not all imagine or think about the same person in the health state when considering the “other” perspectives. Regarding the child, some thought about their own child or a child in their family; others thought about children they had experience with, particularly in ill health; and others thought about a hypothetical child. For another adult, many participants thought about an older family member, particularly one who had experienced ill health. Further, participants’ also imagined the impact of ill health on a person’s extended social circle, which would differ depending on who was imagined. The implication of this is that taking the perspective of others without specifying who this other adult is (e.g. “somebody like you”, “an average citizen”, etc.) may introduce additional heterogeneity in elicited preferences, and further that the impact will differ across participants, depending on who is imagined, for example, as a function of parental status or prior experiences with people in ill health. Indeed, it has been found elsewhere [32] that parental status impacts on preferences for adult health states (valued for themselves, i.e. own—adult), and hence it may be that this is a factor that should be taken into account in all valuation studies. Furthermore, if the goal is to standardise who participants’ think about when using perspectives of others in elicitation tasks, then a more detailed specification of who that is needs to be provided.

Further difficulties were noted with taking the perspective of others, and this is important to note since many studies compare utilities elicited using the own—adult and other—child perspective without noting that this involves a change from own to other *and* a change from adult to child (for an exception see [19]). Whilst some participants made the same decision for another adult as they made for themselves, other participants made a different decision because they were less willing to trade life-years for the other adult as they imagined the other adult was a close family member and they wanted the other adult alive due to their relationship with them. Many participants raised that they felt uncomfortable ‘playing God’ and making decisions on behalf of another, whether that other is a child or another adult. Some participants felt that the use of any other perspective—child or adult—meant that they would not trade years of life in the same way as they would for themselves, since they felt more comfortable about saying what they would prefer for themselves but not what they would prefer for others. This is consistent with recent studies, including a survey finding that adults were more willing to trade life years for themselves than for their child or elderly parent [19, 33].

The perspective of themselves as a child raised the issue around whether they were living now or in the time period when they were a child, citing that this would make a difference since technology and society may be different now meaning that the impact on their lives would differ. Participants expressed that this was an odd question to ask.

Our results indicate that the use of own—adult perspective in health state valuation studies (with adults valuing child and adolescent health states for themselves) makes the elicited preferences more likely to be independent from participants’ parental status or prior experience and emotional investment with children in ill health. However, it should be noted that preferences for own health, and hence valuation studies using the own—adult perspective, may be impacted by consideration upon the impact and burden on others (see for example [34]). Participants found that taking another perspective, and particularly the other—child perspective, meant that the decision was more difficult (also found elsewhere [35]) and that their answers may include emotion and inclusion of other factors, confounded by whom the participants imagined was in those health states. The choice of which perspective, given this, remains a normative decision that should take into account the application of the elicited values to inform resource allocation decisions.

Our findings are of relevance for the valuation of generic child- and adolescent-specific measures such as CHU9D [36] and EQ-5D-Y-3L [5, 6, 37] and EQ-5D-Y-5L [38], but also for the valuation of vignettes for children and adolescents [39] and condition-specific preference-based measures developed for use in children and adolescents [40]. Our findings have implications for the international valuation protocol of the EQ-5D-Y-3L [41] that recommends the elicitation of preferences (using DCE with no duration attribute and TTO) based on a child perspective of a 10 year old child, phrased as “considering your views for a 10-year old child”. Our results indicate that the values generated using this protocol may be heterogeneous due to differences in whom the participants valuing the health states imagine and that the results may be impacted by the composition of the sample, in particular, with regard to the proportion of parents of children aged below 18 [32]. Further, the findings imply that valuation is likely to change if undertaken using an adolescent perspective instead of a child perspective (or for a child of a different age), though further research on this issue is recommended. Ultimately, this study is one source of evidence that can be considered by researchers alongside other sources in making informed decisions for the valuation protocol of a child health measure.

The study found that many participants did not think children should be prioritised in healthcare resource allocation over working-age adults, and whilst there was no consensus, many participants stated that equal prioritisation should be given to all regardless of age. This is consistent with the ‘A QALY is A QALY is A QALY’ view that is usually taken to inform healthcare resource allocation decisions and that applies the same threshold to assess whether a new treatment is recommended for use for either children or adults. However, this may be contrary to some decisions that are made in healthcare [42] and to a recent general population survey [43]. Indeed, support was found a “fair innings” argument; many participants thought that a younger person should be prioritised over an elderly person (e.g. 70 years plus) for healthcare in a forced choice scenario, even with the gain fixed.

## Limitations

Study limitations include that the sample was localised and may suffer from a lack of breadth, meaning that whilst we reached saturation in the data that was collected, it is possible that not all potential themes were raised in our focus groups. However, the sample was purposively recruited to have a mix of gender, age, ethnicity, parental status and education. Our sample had more health problems in pain or discomfort and being worried, sad or unhappy than other dimensions, but this is normal in nationally representative samples [44].

Also of note is that the exercises were paper-based and conducted individually in a group setting, and this differs to many valuation studies that elicit preferences on a one-on-one basis using a computer-assisted personal interview. The perspectives were completed in a fixed order and so we cannot discount the potential influence of an “order effect” on some of our findings, although this is more likely to be relevant to the quantitative than qualitative data. Further, we cannot rule out the possibility that some of the themes emerging from this analysis were due to methodological choices made by the researchers, including the use of dichotomised health states aligned to mental versus physical health problems; not specifying an age for the other—adult perspective; and not following a full interview protocol in the example valuation exercises, including not presenting the TTO valuation task for states worse than dead. The latter may also have impacted on the quantitative results presented in Table 1.

**Table 1:** Time trade-off utilities and discrete choice experiment choices

Perspective	First 3 focus groups, valued moderate health states (*n* = 15)		Last 3 focus groups, valued severe health states (*n* = 15)	
	Mean (*SD*) TTO value	% DCE choosing each state	Mean (*SD*) TTO value	% DCE choosing each state
*State A*				
	State 11223		State 11333	
Own—adult	0.73 (0.25)	26.7	0.55 (0.28)	60.0
Other—adult	0.69 (0.32)	26.7	0.59 (0.25)	26.7
Other—child	0.74 (0.23)	40.0	0.69 (0.26)	6.7
Own – child	0.63 (0.31)	46.7	0.67 (0.27)	20
*State B*				
	State 22311		State 33311	
Own—adult	0.74 (0.24)	73.3	0.58 (0.26)	40.0
Other—adult	0.78 (0.29)	73.3	0.65 (0.31)	73.3
Other—child	0.80 (0.23)	60.0	0.87 (0.21)	93.3
Own—child	0.72 (0.28)	53.3	0.67 (0.32)	80

*TTO* time trade-off, *DCE* discrete choice experiment. TTO values ranged from 0 to 1

Another study limitation is that the semi-structured discussion around the prioritisation of adult versus child health did not involve full systematic consideration of opportunity cost and cost-effectiveness analysis arguments, but was based upon a lay discussion of participants’ views. It could also be argued that the focus group methodology has led to some of the themes being discussed more generally than facilitating a systematic, point-by-point comparison across each perspective, which may be achieved with an alternative method (e.g. think-aloud interviews). Further research is encouraged exploring these and related issues.

## Conclusions

In conclusion, our findings reinforce that if an adult sample is used to value child- and adolescent-specific health states it is important to consider methodologically the impact of the perspective that is used. Whilst choice of perspective is a normative issue, its impact on utility values must be taken into account in making decisions about perspective, as members of the adult general population provide different responses when different perspectives are used due to differences in the perceived impact on utility. Our findings highlight that imagining a 10-year old child is difficult and there is variation in who is imagined by participants. The dimensions being valued may also be relevant, as in our study adults were more willing to give up similar life years for children as adults in situations involving mental suffering and pain. If adults are asked to imagine a child we recommend that sampling is representative for parental status of children aged below 18, since this can impact on preferences. The choice of the age of the child to choose should also involve consideration since some participants will think of the exact age of the child and transitional life stages (e.g. 8 years until a 10 year old becomes an adult). Overall, we did not find support for prioritising child health over working-age adult health, but there was support for a “fair innings” argument.

## Supplementary Information

**Additional file 1**. Appendix A–D.

## Data Availability

The datasets used and/or analysed during the current study are available from the corresponding author on reasonable request.

## Abbreviations

CHU9D: Child Health Utility 9D
DCE: Discrete choice experiment
MVH: Measurement and valuation of health
NICE: National Institute for Health and Care Excellence
PBAC: Pharmaceutical Benefits Advisory Committee
QALY: Quality-adjusted life year
TTO: Time-trade-off
VAS: Visual analogue scale
