# Supplementary material for: Valuing child and adolescent health: a qualitative study on different perspectives and priorities taken by the adult general public
Source: Health Qual Life Outcomes. 2021 Sep 23;19:222. doi: 10.1186/s12955-021-01858-x (PMC8461831; doi:10.1186/s12955-021-01858-x)
Supplement: Supplementary file 1 — Additional file 1. Appendix A–D. [file 12955_2021_1858_MOESM1_ESM.docx]

Appendix

Appendix A. Example of the paper-based booklet used in the focus groups

This example is taken from the first 3 focus groups where respondents valued mild health states, and asks respondents to complete the tasks from their own perspective.


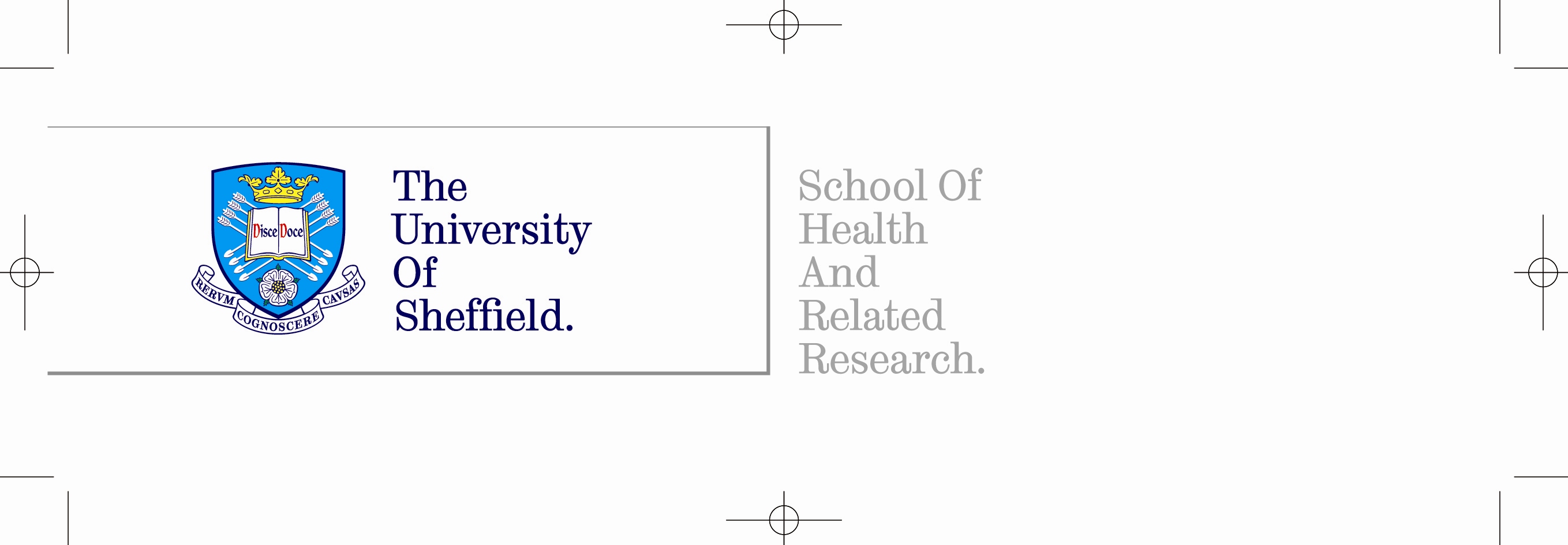


**University of Sheffield**

**School of Health and Related Research**

**Assessing the impact of perspectives and priorities on the valuation of health benefits**

**Questionnaire 1 of 4**

**This questionnaire contains questions which ask about aspects of your health and about you.**

**You will then be presented with a description of ill health, a health state. We will ask you how good or bad you think living in that health state would be. You will then answer the same question for a different health state. You will then be asked which of the two health states you prefer.**

**Please read each question and consider your answers carefully. There are no right or wrong answers; what we want is your opinion.**

**It is important to us that you answer these questions as truthfully and honestly as possible during the whole focus group. This will allow researchers to make the most use of your responses to potentially improve methods used to enhance health services.**

**Thank you for completing this survey.**

Please answer the following information about yourself.

1. **Are you:**

Male [ ]

Female [ ]

|  |
| --- |

**2. What is your age (in years)?**

**3. Do you have any children aged 18 years or younger?**

Yes [ ]

No [ ]

**Describing your health today**

Under each heading, please tick the ONE box that best describes your health TODAY

| **4. Mobility** *(walking about)* |  |
| --- | --- |
| I have no problems walking about | \|  \| \| --- \| |
| I have some problems walking about | \|  \| \| --- \| |
| I have a lot of problems walking about | \|  \| \| --- \| |
| **5. Looking after myself** |  |
| I have no problems washing or dressing myself | \|  \| \| --- \| |
| I have some problems washing or dressing myself | \|  \| \| --- \| |
| I have a lot of problems washing or dressing myself | \|  \| \| --- \| |
| **6. Doing usual activities** *(for example, hobbies, sports, doing things with family or friends)* |  |
| I have no problems doing my usual activities | \|  \| \| --- \| |
| I have some problems doing my usual activities | \|  \| \| --- \| |
| I have a lot of problems doing my usual activities | \|  \| \| --- \| |
| **7. Having pain or discomfort** |  |
| I have no pain or discomfort | \|  \| \| --- \| |
| I have some pain or discomfort | \|  \| \| --- \| |
| I have a lot of pain or discomfort | \|  \| \| --- \| |
| **8. Feeling worried, sad or unhappy** |  |
| I am not worried, sad or unhappy | \|  \| \| --- \| |
| I am a bit worried, sad or unhappy | \|  \| \| --- \| |
| I am very worried, sad or unhappy | \|  \| \| --- \| |

Living life in ill health

Now we want to know how good or bad you think living in ill health would be.

Please imagine that **you** are living in the scenario of ill health.

### Question 9

### Health state A

Imagine you, yourself, were in this health scenario and this health scenario was starting today and lasting for 10 years. After the 10 years you will die.

| - I have no problems walking about - I have no problems washing or dressing myself - I have a lot of problems doing my usual activities - I have a lot of pain or discomfort - I am very worried, sad or unhappy |
| --- |

Please put **A** in each row where you would rather live in **state A**, described above, for 10 years and then die.

Please put **F** in each row where you would rather live in **full health** for the number of years shown and then die.

Please put an ‘**=**’ in each row where you **cannot choose** between state A for 10 years and full health for the number of years shown.

| **State A** | **Please indicate your response below** | **Full health** |
| --- | --- | --- |
| 10 years |  | 10 years |
| 10 years |  | 9 years 6 months |
| 10 years |  | 9 years |
| 10 years |  | 8 years 6 months |
| 10 years |  | 8 years |
| 10 years |  | 7 years 6 months |
| 10 years |  | 7 years |
| 10 years |  | 6 years 6 months |
| 10 years |  | 6 years |
| 10 years |  | 5 years 6 months |
| 10 years |  | 5 years |
| 10 years |  | 4 years 6 months |
| 10 years |  | 4 years |
| 10 years |  | 3 years 6 months |
| 10 years |  | 3 years |
| 10 years |  | 2 years 6 months |
| 10 years |  | 2 years |
| 10 years |  | 1 years 6 months |
| 10 years |  | 1 years |
| 10 years |  | 6 months |
| 10 years |  | 0 years |

**Question 10**

### Health state B

Imagine you, yourself, were in this health scenario and this health scenario was starting today.

| - I have a lot of problems walking about - I have a lot of problems washing or dressing myself - I have a lot of problems doing my usual activities - I have no pain or discomfort - I am not worried, sad or unhappy |
| --- |

Please put **B** in each row where you would rather live in **state B**, described above, for 10 years and then die.

Please put an **F** in each row where you would rather live in **full health** for the number of years shown and then die.

Please put an ‘**=**’ in each row where you **cannot choose** between state B for 10 years and full health for the number of years shown.

| **State B** | **Please indicate your response below** | **Full health** |
| --- | --- | --- |
| 10 years |  | 10 years |
| 10 years |  | 9 years 6 months |
| 10 years |  | 9 years |
| 10 years |  | 8 years 6 months |
| 10 years |  | 8 years |
| 10 years |  | 7 years 6 months |
| 10 years |  | 7 years |
| 10 years |  | 6 years 6 months |
| 10 years |  | 6 years |
| 10 years |  | 5 years 6 months |
| 10 years |  | 5 years |
| 10 years |  | 4 years 6 months |
| 10 years |  | 4 years |
| 10 years |  | 3 years 6 months |
| 10 years |  | 3 years |
| 10 years |  | 2 years 6 months |
| 10 years |  | 2 years |
| 10 years |  | 1 years 6 months |
| 10 years |  | 1 years |
| 10 years |  | 6 months |
| 10 years |  | 0 years |

Question 11

Comparing health states A and B

Please imagine you live for 10 years in either description of health, and then you die.

| **Health state A** | **Health state B** |
| --- | --- |
| I have no problems walking about | I have a lot of problems walking about |
| I have no problems washing or dressing myself | I have a lot of problems washing or dressing myself |
| I have a lot of problems doing my usual activities | I have a lot of problems doing my usual activities |
| I have a lot of pain or discomfort | I have no pain or discomfort |
| I am very worried, sad or unhappy | I am not worried, sad or unhappy |
| **Which do you prefer?**  A B | |

**Appendix B. Indicative semi-structured topic guide**

| **Stage of focus group** | **Moderator guidelines** |
| --- | --- |
| Introduction | Provide and discuss information sheet and consent form  Ask permission to audio record |
| Explain background of research | There is a lot of interest in understanding how important different aspects of health are. Understanding what people think is important and may be used to make decisions on how to allocate money in the NHS and for some public services. The overall aim of this project is to find out what people think about when considering how important different aspects of health are.  We will ask you a series of questions that involve imagining you or somebody else living in certain health states.  The moderator will briefly explain:  The aims of the research – is to find out what people think about when they think about living with ill health and how good or bad their life would be. With this study, we would like to better understand what people think about when they imagine themselves or others living in ill health and the impact on their life.  To start the focus group we will ask you to answer 4 brief questionnaires, which you will all answer individually. There are questions that ask you to think about how good or bad different scenarios of ill health are, and ask you which ill health scenarios you think are best or worst. Some questions will ask you to imagine you are living in ill health, and other questions will ask you to imagine somebody else living in ill health.  When you have all answered the 4 questionnaires we will then have a group discussion.  The group discussion will involve talking about what you thought about when answering the questions. You will also be asked how you think money that is spent on the NHS should be allocated across people of different ages. You will all discuss your responses and views together with all of the other members of the focus group. |
| Answering 4 short questionnaires | Please complete questionnaire 1 now.  After all members of the group have completed questionnaire 1, they will be asked to complete questionnaire 2.  After all members of the group have completed questionnaire 2, they will be asked to complete questionnaire 3.  After all members of the group have completed questionnaire 3, they will be asked to complete questionnaire 4. |
| Stage 1 of focus group discussion: Exploring understanding and interpretation of health states | *This stage will explore:*   1. *Participants’ understanding and interpretation of the health states and the valuation tasks;* 2. *What they thought about when providing their values, for example, the consequences of the health states* 3. **Do you think you understood what it would be like to live in the health states?**   Were the health states easy or difficult to understand? Why was that?  Were you able to imagine yourself in that health state? Was it difficult or easy to do that? Why?  Interpretation of the dimensions of the EQ-5D, did they focus on certain words of the description of the health state?   1. **What did you think about when you were trying to imagine living in the health states?**   How did you go about imagining living with ill health?  What kind of things were you imagining (e.g. consequences such as independence, impact on others…)?  What were your main considerations when giving your answers? For example, were there any important life events or circumstances driving your responses?  Did any of you draw on personal experiences or experiences of your family/friends? |
| Stage 2 of focus group: Exploring differences by perspective and task | *This stage will explore:*   1. *Potential differences in responses provided in the TTO and DCE tasks, and when the health state was experienced by the participant themselves, another adult, another child, or themselves as a child;* 2. *The reasoning behind any differences or similarities in the values, for example: whether potential differences are around differential impact on the health or quality of life of yourself versus another adult, another child or yourself as a child; differential impact on what you would be prepared to sacrifice on behalf of another person; judgements around whether you wish to avoid suffering of other adults or a child in comparison to yourself;* 3. **Did you answer differently when the questions changed who was experiencing the ill health, that is whether it was experienced by yourselves, another adult, a 10-year-old child, or yourself as a child?**   If you did not answer differently, do you think you might have answered differently for different health states?   1. **Why did you answer differently (or why did you not answer differently)?**   Did you change what you were thinking about when we changed who was experiencing the health state?  Did you find it easier or harder to answer when we changed who was experiencing the health state?  Were you willing to trade years of life for somebody else?   1. **What about the different tasks – the first tasks where you traded years of life and the task where you chose which health state was best? Did your answers change in the same way for all of the tasks?**   Are you surprised by any of the differences?  What do you think explains any of the differences?  Which set of views do you think is more accurate? And why?  Do you think the differences are problematic? |
| Stage 3 of focus group: Exploring solutions | *This stage will explore:*   1. *Whether participants feel their general attitudes around child and adult health and the prioritisation of child versus adult health influenced their values; and* 2. *Which values participants think are most appropriate for informing public policy.* 3. **Do you think the health of children is more important than the health of adults (or vice versa)? Did your views on this affect your answers to the questions?**   Do you think the NHS should prioritise the treatment of children and adolescents rather than adults?  Do you think the NHS should be prepared to pay a higher amount to pay to treat children rather than adults?   1. **In the UK we make decisions about how money in healthcare is spent using what the public think about health states. Some health states are experienced by children, but the public don’t always know that we ask then what they think about the health states. . When we ask the questions about what people think about health states, should we tell them if they are experienced by children?**   What do you think we should do?  How do you think we should ask the questions?  Do you have any other comments you would like to make? |
| Conclude | Thank participants for their participation and ask for completed questionnaires |

**Appendix C. Framework and coverage of themes**

|  |  |  | **Theme coverage*** | | |
| --- | --- | --- | --- | --- | --- |
| **Focus group topic** | **Category** | **Theme** | **References** | **Participants** | **Groups** |
| Interpreting the health states | Understanding health states | Ease of imagining health states | 17 | 15 | 6 |
|  |  | Implausible combinations | 10 | 8 | 3 |
|  |  | Interpretation of health state severity | 24 | 18 | 5 |
|  |  | Anticipated change or adaptation | 24 | 22 | 6 |
|  |  | Drawing on personal experience | 40 | 22 | 6 |
|  |  | Individual characteristics | 7 | 7 | 4 |
|  | Relative importance of health state dimension | Mental vs. physical health | 38 | 24 | 6 |
|  |  | Importance of individual dimensions | 14 | 12 | 5 |
|  | Consequences of ill health | Impacting others | 33 | 19 | 5 |
|  |  | Impacting activities | 5 | 8 | 5 |
|  |  | Maintaining dignity | 6 | 5 | 2 |
| Differences by perspective and task | Perceived differences between adults and children | Generational differences | 8 | 11 | 3 |
|  |  | Child vs adult ill health | 54 | 23 | 6 |
|  |  | Willingness to trade for children | 42 | 25 | 6 |
|  | Considerations when deciding for others | Putting self in others' shoes | 6 | 7 | 5 |
|  |  | Deciding for self vs. others | 25 | 19 | 5 |
|  |  | Who is imagined | 51 | 27 | 6 |
|  |  | Difficulty playing God | 24 | 18 | 6 |
|  |  | Taking age into account | 13 | 11 | 5 |
|  |  | Giving people a life worth living | 30 | 17 | 6 |
|  | Views on elicitation technique | Prefer DCE or TTO | 18 | 19 | 6 |
|  |  | Perspective changes within task | 7 | 10 | 5 |
| Exploring solutions | Prioritising healthcare | Prioritising by age | 68 | 27 | 6 |
|  |  | Prioritising by other criteria | 22 | 25 | 6 |
|  |  | Case by case decision | 5 | 8 | 4 |
|  | Being informed | Knowing who is in ill health | 23 | 25 | 6 |
|  |  | Changing responses following discussion | 9 | 7 | 3 |

*Note*. *Theme coverage is an estimate of the frequency that the theme occurred during the focus group discussions. ‘References’ refers to instances that the theme was coded; ‘Participants’ is the number of people that appeared to engage (however briefly) in the discussion on that theme; and ‘Groups’ is the number of focus groups where that topic was discussed.

**Appendix D. Data saturation matrix**

|  | **Focus group where theme was indexed** | | | | | |
| --- | --- | --- | --- | --- | --- | --- |
| **Theme** | **1** | **2** | **3** | **4** | **5** | **6** |
| Anticipated change or adaptation | X | X | X | X | X | X |
| Case by case decision | X | X |  | X |  | X |
| Changing responses following discussion |  | X | X |  |  | X |
| Child vs adult ill health | X | X | X | X | X | X |
| Deciding for self vs. others |  | X | X | X | X | X |
| Difficulty playing God | X | X | X | X | X | X |
| Drawing on personal experience | X | X | X | X | X | X |
| Ease of imagining health states | X | X | X | X | X | X |
| Generational differences | X |  |  |  | X | X |
| Giving people a life worth living | X | X | X | X | X | X |
| Impacting activities | X | X | X | X | X |  |
| Impacting others | X | X |  | X | X | X |
| Implausible combinations | X |  | X |  | X |  |
| Importance of individual dimensions | X | X |  | X | X | X |
| Individual characteristics | X |  |  | X | X | X |
| Interpretation of health state severity | X | X | X | X | X |  |
| Knowing who is in ill health | X | X | X | X | X | X |
| Maintaining dignity |  |  |  | X | X |  |
| Mental vs. physical health | X | X | X | X | X | X |
| Perspective changes within task | X | X | X | X | X |  |
| Prefer DCE or TTO | X | X | X | X | X | X |
| Prioritising by age | X | X | X | X | X | X |
| Prioritising by other criteria | X | X | X | X | X | X |
| Putting self in others' shoes | X | X |  | X | X | X |
| Taking age into account | X | X | X | X |  | X |
| Who is imagined | X | X | X | X | X | X |
| Willingness to trade for children | X | X | X | X | X | X |

*Note*. Themes are listed alphabetically (not within categories).
